# Identifying Problematic Subjects Entering Clinical Trials in Early Alzheimer’s Disease

**DOI:** 10.1002/alz70859_106781

**Published:** 2025-12-26

**Authors:** Alan Kott, Xingmei Wang, David S. Miller

**Affiliations:** ^1^ Signant Health, Prague Czech Republic; ^2^ Signant Health, Blue Bell, PA USA

## Abstract

**Background:**

Our prior retrospective analyses found that a large portion of subjects randomized into early Alzheimer's Disease trials were more severe than required by the protocol.

We have identified a number of individual factors that can help recognize these subjects at the time of screening into the clinical trial.

In this retrospective analysis, we tested the performance of these factors, both alone and in combination, to distinguish these subjects.

**Method:**

Using logistic regression, we tested the performance of all possible combinations of 3 dichotomous risk factors (screening MMSE interview duration length, screening MMSE score and screening performance on confirmatory cognitive tests (RBANS, FCSRT, WMS‐LMII, etc.)) to predict at screening, those subjects whose MMSE scores will fall below the inclusionary threshold at baseline. All analyzes were performed on screening and baseline data obtained from 1,966 subjects randomized into early AD clinical trials.

**Result:**

Compared to the situation when none of the 3 individual factors triggered, the odds of the subject being more severe were significantly increased in all other combinations of the 3 factors. Having only 1 risk factor present increased the odds between 2 to 8 times, having 2 risk factors present increased the odds between 7 to 13 times, and finally, having all 3 risk factors present increased the odds 22 times.

**Conclusion:**

In this retrospective analysis we have tested the performance of 3 risk factors – alone and in combination ‐ to recognize subjects who are likely more severe than required by the protocol. Subjects with screening MMSE scores close to the inclusionary thresholds, with longer screening MMSE interviews and poor performance on confirmatory cognitive scales were 22x more likely to be deemed more severe than the inclusion criteria stipulated at baseline than those subjects who did not have any of the 3 risk factors. This finding is important as it allows researchers to identify and possibly screen fail these subjects before they get randomized, thus preserving the integrity of the clinical trial. Among the limitations of this analysis is its retrospective nature and narrow focus, predominantly on MMSE data.

